# Photoelectrochemical Immunosensor for Detection of Carcinoembryonic Antigen Based on 2D TiO_2_ Nanosheets and Carboxylated Graphitic Carbon Nitride

**DOI:** 10.1038/srep27385

**Published:** 2016-06-06

**Authors:** Huan Wang, Yaoguang Wang, Yong Zhang, Qi Wang, Xiang Ren, Dan Wu, Qin Wei

**Affiliations:** 1 Key Laboratory of Chemical Sensing & Analysis in Universities of Shandong, School of Chemistry and Chemical Engineering, University of Jinan, Jinan 250022, China; 2 School of Material Science and Engineering, University of Jinan, Jinan 250022, P.R. China

## Abstract

Carcinoembryonic antigen (CEA) was used as the model, an ultrasensitive label-free photoelectrochemical immunosensor was developed using 2D TiO_2_ nanosheets and carboxylated graphitic carbon nitride (g-C_3_N_4_) as photoactive materials and ascorbic acid as an efficient electron donor. 2D TiO_2_ nanosheets was sythsized by surfactant self-assembly method and proved to have higher photoelectrochemical signals than TiO_2_ nanoparticles. Firstly, carboxylated g-C_3_N_4_ could be attached to 2D TiO_2_ nanosheets through the bond formed between carboxyl group of carboxylated g-C_3_N_4_ and TiO_2_. And the photocurrent of g-C_3_N_4_/TiO_2_ drastically enhances compared to carboxylated g-C_3_N_4_ and TiO_2_. Then, antibody of CEA was bonded to TiO_2_ through the dentate bond formed between carboxyl group of anti-CEA and TiO_2_, leading to the decrease of the photocurrents. As proven by PEC experiments and electrochemical impedance spectroscopy (EIS) analysis, the fabrication process of the immunosensor is successful. Under the optimal conditions, the intensity decreased linearly with CEA concentration in the range of 0.01~10 ng/mL. The detection limit is 2.1 pg/mL. The work provides an effective method for the detection of tumor markers and can be extended for the application in food safety and environmental monitoring analysis.

Photoelectrochemical (PEC) sensors are fabricated on photoactive electrodes which can convert photoirradiation to electrical signal. Therefore, photoactive materials are crucial for the performance of the PEC sensors. Many photoactive materials are metal-contained semiconductors, such as TiO_2_, CdSe, CdTe, ZnO, ZnS, etc^1^,^2^,^3^,^4^,^5^,^6^,^7^,^8^,^9^. Among them, titanium dioxide is one of the most commonly employed materials because of its nontoxicity, low-cost and brilliant photochemical and chemical stability. Nevertheless, due to a wide energy band gap of 3.0 eV, TiO_2_ can only absorb the ultraviolet light (<387 nm)^10^. So applications of pure TiO_2_ are also limited. Another class of photoactive materials is metal-free polymeric semiconductor. Graphitic carbon nitride (g-C_3_N_4_), a moderate energy bandgap of 2.7 eV, has paid more and more attentions because it is very stable in acid or alkaline electrolytes^11^. However, applications of pure g-C_3_N_4_ are also limited largely because of its low quantum efficiency and high electron−hole recombination rate. As mentioned above, researchers have therefore made great efforts to expand the application of pure TiO_2_ and pure g-C_3_N_4_ by various methods, such as coupling it with other materials, nanostructuring and doping^12^,^13^. Thus, owing to the proper band level between g-C_3_N_4_ and TiO_2_, g-C_3_N_4_ and TiO_2_ are combined together leading to the easy separation of the photo-generated electron and hole. Although there are some references about the combination of g-C_3_N_4_ and TiO_2_, the morphology of TiO_2_ is maily focused on nanorod^10^,^14^, nanoparticles^11^ and nanotube^15^,^16^,^17^.

Herein, two-dimensional (2D) TiO_2_ nanosheets was prepared by forming inverse lamellar micelles of Pluronic P123 surfactant together with ethylene glycol (EG) co-surfactant in ethanol solvent. It is reported that 2D nanostructures exhibit superior catalytic, photovoltaic and electrochemical performances, due to their large surface-to-volume ratio and confined thickness on the atomic scale^18^. So they could have promising applications in sensors, and energy conversion and storage devices. Using carcinoembryonic antigen (CEA) as a model analyte, we developed a label-free photoelectrochemical immunosensor for detection of CEA based on 2D TiO_2_ nanosheets and carboxylated graphitic carbon nitride. CEA, a usual tumor marker, can be used for the early detection of recurrent diseases and indicate the effect of therapy in early breast cancer and gastrointestinal cancers as well as other tumor markers^19^,^20^. Coupling carboxylated graphitic carbon nitride with 2D TiO_2_ nanosheets can evidently extend the absorption range, increase the utilization of light energy, and promote the photocurrent intensity. Then, antibody of CEA was immobilized through the dentate bond formed between carboxyl group of anti-CEA and TiO_2_, leading to the decrease of the PEC signal. The decreased signal is proportional to the logarithm of CEA concentration. The CEA immunosensor exhibits high sensitivity, good selectivity and wide linear range.

## Experimental

### Materials and reagents

Titanium isopropoxide (TTIP, 95%), ethylene glycol (EG) and ascorbic acid were purchased from Sinopharm Chemical Reagent Co., Ltd. (China). The CEA and corresponding antibody were purchased from Beijing Dingguo Changsheng Biotechnology Co. Ltd. (China). Bovine serum albumin (BSA, 96–99%) were purchased from Sigma-Aldrich (Beijing, China). All other chemicals were of analytical grade and used without further purification. ITO glass (resistivity 10 Ω/sq) is obtained from Zhuhai Kaivo Electronic Components Co. Ltd., China.

### Apparatus

The scanning electron microscope (SEM) images were obtained by the field emission SEM (ZEISS, Germany). Photoelectrochemical (PEC) measurements were performed on an electrochemical workstation (Zahner Zennium PP211, Germany).

### Synthesis of TiO_2_ nanosheet

TiO_2_ was prepared according to the literature^18^,^21^. 1.05 g TTIP was added into 0.74 g concentrated HCl solution under vigorous stirring (solution A); and 0.2 g Pluronic P123 was dissolved in 3.0 g ethanol (solution B) under stirring for 15 min. Then, solution B was added into solution A and stirred for another 30 min. Subsequently, 2.5 mL TTIP solution with 20 mL EG was transferred into an autoclave and heated at 150 °C for 20 h. After cooled to room temperature naturally, the resulting solid powder was collected by centrifugation and washed with ethanol several times. The final products were then dried at 80 °C for 24 h.

### Synthesis of carboxylated g-C_3_N_4_

Carboxylated g-C_3_N_4_ was prepared as described previously with our reference^22^. In brief, 5.0 g of white melamine powder was put into a covered ceramic crucible and heated at 550 °C for 4 h in a muffle furnace. The yellow g-C_3_N_4_ product was ground to powder after cooling to room temperature. Then, 1 g g-C_3_N_4_ powder was placed into 100 mL 5 mol/L HNO_3_ and refluxed for 24 h at 125 °C. After cooling to room temperature, the refluxed product was centrifuged and washed with water until pH reached 7. Finally, the resulting product was dried at 35 °C for 12 h in vacuum.

### Synthesis of carboxylated g-C_3_N_4_/TiO_2_ solution

In brief, 0.5 mg carboxylated g-C_3_N_4_ powder was dispersed in 5 mL water by ultrasonication for over 2 h. Then, 20 mg TiO_2_ powder was added to the above suspension and stirred for 24 h. After that, the solution was centrifugated and the products were redispersed in 5 mL of water.

### Fabrication of PEC immunosensor

**Figure 1:**
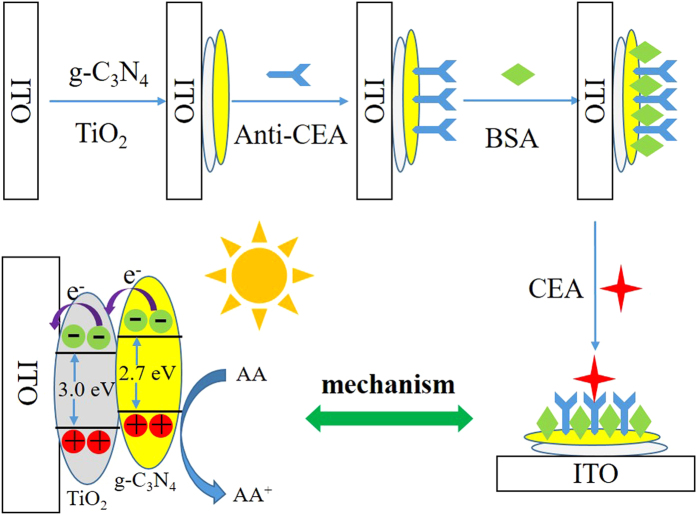


The illustration of PEC immunosensor fabrication process is depicted in Fig. 1. ITO slices (3 × 0.5 cm^2^) were sonicated in acetone, ethanol and water consecutively for 30 min and dried under a N_2_ stream. 6 μL of carboxylated g-C_3_N_4_/TiO_2_ solution was dropped on the surface of the pre-cleaned ITO and dried after air drying, the film was sintered at 450 °C for 30 min and then cooled down to the room temperature. Then, 4 μL of 10 μg/mL anti-CEA solution was bonded onto TiO_2_ for 1 h via the bond formed between carboxyl group of anti-CEA and TiO_2_ at 4 °C for 1 h^23^,^24^. The unreacted active sites on the electrode surface were deactivated by 6 μL of 1% bovine serum albumin (BSA) solution for 1 h. Finally, 6 μL of CEA solutions with different concentrations were incubated for 1 h the electrode was incubated with different concentration of CEA for 1 h min at 4 °C and then washed with buffer solution to remove the excess CEA. Thus, the PEC immunosensor was fabricated completely and was ready to be used.

### PEC measurements

Photocurrent was measured by the current–time curve experimental technique on a photoelectrochemical workstation at a bias voltage of 0.1 V with light intensity of 150 W/cm^2^. All experiments were carried out using a conventional three electrodes system with a modified ITO as working electrode, a Pt wire as counter electrode, and a saturated Ag/AgCl electrode as reference electrode.

## Results and Discussion

### Characterization of TiO_2_ nanosheet

**Figure 2:**
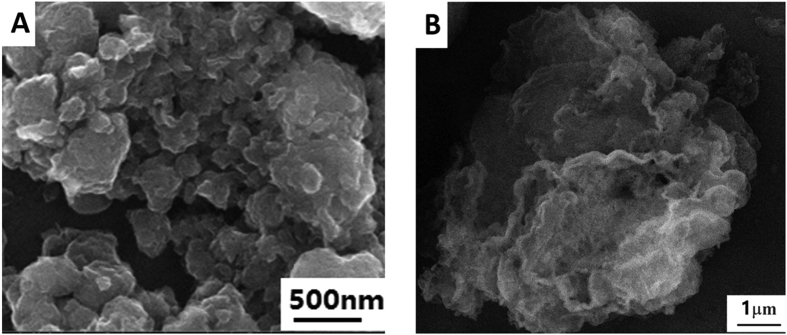
SEM of TiO_2_ nanosheets (**A**) and carboxylated g-C_3_N_4_ (**B**).

SEM images were used to confirm the successful synthesis of nanomaterials with different morphology. Figure 2 show the SEM images of TiO_2_ (A) and carboxylated g-C_3_N_4_ (B). Obviously, TiO_2_ exists in the form of groups of nanosheets. And the prepared carboxylated g-C_3_N_4_ also has the nanosheet structure.

### Characterization of the immunosensor

**Figure 3:**
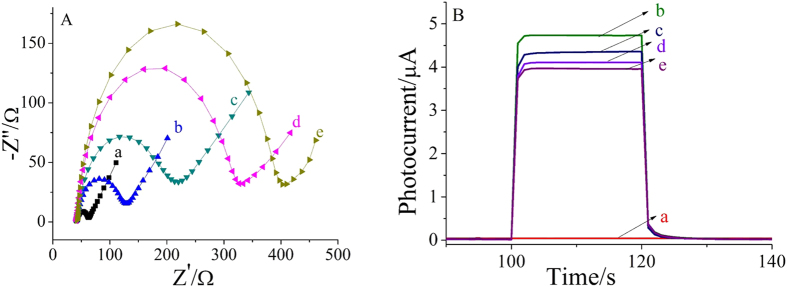
EIS in the presence of 5.0 mmol/L [Fe(CN)_6_]^3−/4−^ solution containing 0.1 mol/L KCl (**A**) and photocurrent–time curves in 0.1 mol/L PBS (pH = 7.0) containing 0.1 mmol/L AA with 0.1 V applied potential and 430 nm excitation light. (**B**) (a) ITO (b) ITO/g-C_3_N_4_/TiO_2_ composite (c) ITO/g-C_3_N_4_/TiO_2_/Anti-CEA (d) ITO/g-C_3_N_4_/TiO_2_/Anti-CEA/BSA (e) ITO/g-C_3_N_4_ /TiO_2_/Anti-CEA/BSA/CEA.

Electrochemical impedance spectroscopy (EIS), a useful tool for evaluating electron transfer resistance, was used to monitor the stepwise modification of the electrodes. The semicircle diameter corresponds to the electron transfer resistance (Ret), which reflects the restricted diffusion of the redox probe accessing the layer^25^. The measurements were carried out in 5.0 mmol/L [Fe(CN)_6_]^3−/4−^ solution containing 0.1 mol/L KCl and the result were shown in Fig. 3A. Non-modified ITO electrode showed a small semicircle diameter (curve a), implying a low electron transfer resistance. After coating of carboxylated g-C_3_N_4_/TiO_2_ composite (curve b), semicircle diameter increases gradually because both carboxylated g-C_3_N_4_ and TiO_2_ as semiconductors evidently reduced the ability of the redox probe to access the electrode surface. The sequential immobilization of CEA antibody (curve c), BSA (curve d) and CEA (curve e) led to gradual increase of the electron transfer resistance because of the insulation properties of protein.

In order to further confirm that the electrode was modified successfully, the stepwise fabrication process of the immunosensor was also characterized by PEC measurements, as shown in Fig. 3B. Compared with the ITO (curve a), the photocurrent response (curve b) was enhanced greatly after carboxylated g-C_3_N_4_/TiO_2_ composite was immobilized on it subsequently, suggesting that carboxylated g-C_3_N_4_/TiO_2_ have good PEC properties. With the loading of CEA antibody (curve c), BSA (curve d) and CEA (curve e) onto the modified electrode surface successively, the photocurrent intensity decreased which could be attributed to the block of biomacromolecules. Both the above results were consistent with the fact that the electrode was modified as expected.

The mechanism of electron transfers in g-C_3_N_4_/TiO_2_ PEC immunosensor in ascorbic acid (AA) electrolyte probably is that high electron−hole recombination rate in g-C_3_N_4_ results in low PEC activity, when g-C_3_N_4_/TiO_2_ composites are exposure, the photo-generated electrons from g-C_3_N_4_ can transfer from conduction band of g-C_3_N_4_ to the conduction band of TiO_2_. Because the conduction band and valence band edges of g-C_3_N_4_ are higher than those of TiO_2_ nanosheets, the above transfer process of photo-generated carriers is easy. AA is a kind of excellent electron donor, which could block the recombination of photo-generated electrons and holes and meanwhile promote the electron transfer from conduction band of TiO_2_ nanosheets to the ITO electrode. The specific binding of CEA to its antibody blocked the electron transfer from AA to g-C_3_N_4_/TiO_2_ composite, resulting in the recombination of photo-generated holes and electrons, which could explain a decrease in photocurrent. Moreover, the photocurrent intensity decreased gradually with the increase of CEA concentration. Therefore, the quantitative detection of CEA is achieved by monitoring the photocurrent decrease after the binding of CEA.

### Optimization of experimental conditions

**Figure 4:**
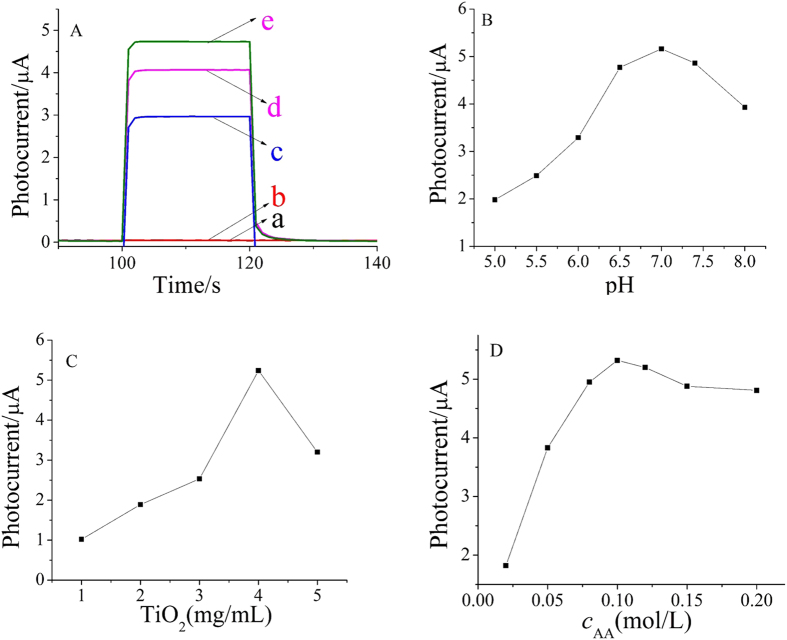
Photocurrent responses of different modified materials (**A**) effect of pH (**B**) the concentration of TiO_2_ (**C**) and the concentration of AA (**D**) on the photocurrent intensity. (a) ITO (b) ITO/g-C_3_N_4_ (c) ITO/TiO_2_ nanoparticles (d) ITO/TiO_2_ nanosheets (e) ITO/g-C_3_N_4_/TiO_2_ composite.

As shown in Fig. 4A, no PEC signal were found for the ITO (curve a). Compared with the ITO, the PEC signal changed only a little after modified with g-C_3_N_4_ (curve b) due to the low quantum efficiency and high electron−hole recombination rate of g-C_3_N_4_. Moreover, TiO_2_ showed obvious PEC signal, and the photocurrent intensity of TiO_2_ nanosheets (curve d) was 30% higher than that of TiO_2_ nanoparticles (curve c). It illustrates that TiO_2_ with different morphology has different photoelectric response performance, and nanosheet is superior to nanoparticles in this system. Although pure g-C_3_N_4_ did not show obvious PEC signal, but when it was combined with TiO_2_, the PEC signal was significantly increased (curve e), which was 12% higher than that of pure TiO_2_ nanosheets and much larger than the sum of the two kinds of nanomaterials, suggesting there is energy level matching between g-C_3_N_4_ and TiO_2_. This is the reason why both carboxylated g-C_3_N_4_ and TiO_2_ were chosen as photoactive materials.

To obtain an optimal PEC signal, pH value of substrate solution was investigated. Keeping the concentrations of CEA constant, the effect of pH on the photocurrent intensity was studied over a pH range from 5.0–8.0, as shown in Fig. 4B. The photocurrent intensity increased with the increase of pH from 5.0–7.0 and reached the maximum. After that, the photocurrent intensity decreased accordingly with pH increasing from 7.0–8.0. This suggests that the neutral condition is more advantageous to the photoelectric response of the carboxylated g-C_3_N_4_ and TiO_2_ nanosheets system. Therefore, pH 7.0 was chosen as the optimal value.

The effect of TiO_2_ concentration was also tested. As shown in Fig. 4C, the photocurrent intensity increased when TiO_2_ concentration increased from 1 to 4 mg/mL because more photoactive materials are formed and light absorption is enhanced. After that, the photocurrent intensity is decreased because thicker C_3_N_4_/TiO_2_ film could also lead to increased diffusion resistance for electron motion. Thus, 4 mg/mL TiO_2_ was chosen for subsequent study.

The concentration of AA as an efficient electron donor was evaluated to improve the photocurrent response of the PEC sensor. It can be seen from Fig. 4D that the photocurrent intensity reached a maximum value at 0.1 mol/L AA. Thus, 0.1 mol/L AA PBS solution (pH 7.0) was used as the buffer electrolyte for CEA detection.

**Figure 5:**
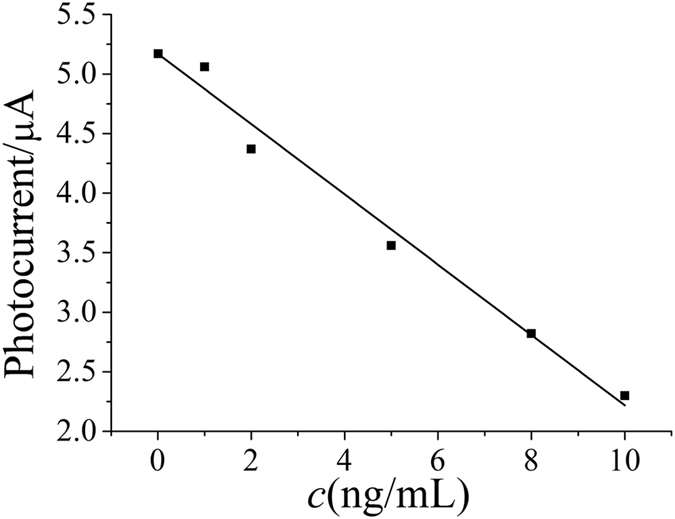


Under the optimal conditions, a series of photoelectrochemical immunosensors were incubated with different concentrations of CEA and subsequent determination was carried out. As expected, the photocurrents decreased with the increase of CEA concentration due to the biomolecular insulation properties of CEA. Moreover, it can be seen from Fig. 5 that the photocurrent intensity decreased linearly with CEA concentration in the range from 0.01–10 ng/mL with a detection limit of 2.1 pg/mL (S/N = 3). Table 1 shows the comparison of the proposed photoelectrochemical immunosensors with other previously reported immunosensors for the detection of CEA^26^,^27^,^28^,^29^,^30^. As seen from the table, the detection limit of this work are superior to the previously reported immunosensors, indicating that the proposed PEC immunosensor has a good performance.

### Stability and Selectivity

**Figure 6:**
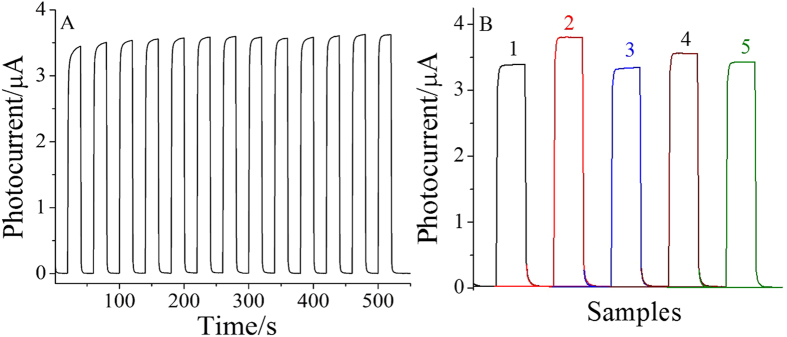
The stability of the immunosensor incubated with 5 ng/mL CEA under several on/off irradiation cycles for 550 s (**A**) and the selectivity of the immunosensor (**B**). 5 ng/mL CEA (1), 5 ng/mL CEA + 50 ng/mL PSA (2), 5 ng/mL CEA + 50 ng/mL H-IgG (3), 5 ng/mL CEA + 50 ng/mL BSA (4), 5 ng/mL CEA + 50 ng/mL glucose (5).

The stability of the designed immunosensor was also evaluated by using a prepared PEC immunosensor for the detection of 5 ng/mL CEA. The photocurrent responses were recorded under several on/off irradiation cycles for 550 s. As shown in Fig. 6A, there was only a little change of the photocurrent, indicating the developed PEC sensors have stable photocurrent response for CEA detection.

Figure 6B shows the selectivity test of the ECL immunosensor for 5 ng/mL CEA. The photocurrents were measured by mixing 5 ng/mL of CEA with 50 ng/mL prostate-specific antigen (PSA), 50 ng/mL human immune globulin G (H-IgG), 50 ng/mL BSA and 50 ng/mL glucose, respectively. The photocurrent exhibited no obvious change compared with the 5 ng/mL of CEA, which indicated excellent selectivity and specificity of the PEC immunosensor for CEA.

### Serum sample analysis

The amount of CEA was measured 5 times in human serum sample and the relative standard deviation (RSD) was calculated to obtain the precision. The accuracy was also studied through a recovery experiment using standard addition method. 1.00 ng/mL CEA standard solution was added to the corresponding samples. With the same experiments measured for five times, the average recovery was calculated. It can be found from Table 2 that the relative standard deviation is 3.1% and the recovery is 99.2%. Hence, the proposed immunosensor can be used for CEA detection with satisfied results.

## Conclusions

This work demonstrated a label-free photoelectrochemical immunosensor for detection of carcinoembryonic antigen using carboxylated g-C_3_N_4_ and 2D TiO_2_ nanosheets as photoactive materials. 2D TiO_2_ nanosheets exhibits better photocatalytic activities than TiO_2_ nanoparticles and g-C_3_N_4_ can improve its photocatalytic performance due to the good energy level matching. Great photocatalytic activities of g-C_3_N_4_/TiO_2_ nanosheets together with the specificity of immunoreaction made sensitive detection of CEA possible. The proposed immunosensor has excellent performance with high sensitivity, good selectivity and stability. Moreover, this strategy could be used to develop photoelectrochemical immunosensors for other targets.

## Additional Information

**How to cite this article**: Wang, H. *et al*. Photoelectrochemical Immunosensor for Detection of Carcinoembryonic Antigen Based on 2D TIO_2_ Nanosheets and Carboxylated Graphitic Carbon Nitride. *Sci. Rep.* **6**, 27385; doi: 10.1038/srep27385 (2016).

## Figures and Tables

**Table 1:** Comparison with other previously reported immunosensors for the detection of CEA.

Materials	Detection ranges (ng/mL)	Detection limits (ng/mL)	References
CS–CNTs–GNPs nanocomposite film	0.1–2.0	0.04	26
[Ag–Ag_2_O]/SiO_2_ nanocomposite material	0.5–160	0.14	27
Thi@NPG/AuNPs	0.01–100	0.003	28
AuNP@nafion/FC@CHIT	0.01–150	0.0031	29
HRP-anti-CEA-NGGN	0.05–350	0.01	30
Carboxylated g-C_3_N_4_/TiO_2_ nanosheets	0.01–10	0.0021	This work

**Table 2:** Results for the determination of CEA in human serum sample.

Sample	Content of PSA (ng/mL)	Average (*n* = 5, ng/mL)	RSD (%)	Added (ng/mL)	Recovery value (ng/mL)	Recovery (*n* = 5, ng/mL)
Human serum	1.35	>1.39	>3.1	1.00	1.03	>99.2
	1.42			1.00	0.95	
	1.45			1.00	1.01	
	1.37			1.00	0.92	
	1.36			1.00	1.05	
